# Chondroid Lipoma Mimicking Malignancy

**DOI:** 10.5334/jbsr.2744

**Published:** 2022-05-05

**Authors:** Kris Mertens, Annelies Van Beeck, Filip Vanhoenacker

**Affiliations:** 1 AZ Sint-Maarten and Catholic University of Leuven, BE; 2 Universital Hospital of Antwerp, BE

**Keywords:** computed tomography, magnetic resonance imaging, conventional radiography, chondroid lipoma

## Abstract

**Teaching Point:** A chondroid lipoma is a rare benign adipocytic neoplasm, containing a chondromyxoid matrix, that may mimic a myxoid liposarcoma and myxoid chondrosarcoma.

## Case History

**Figure 1:**
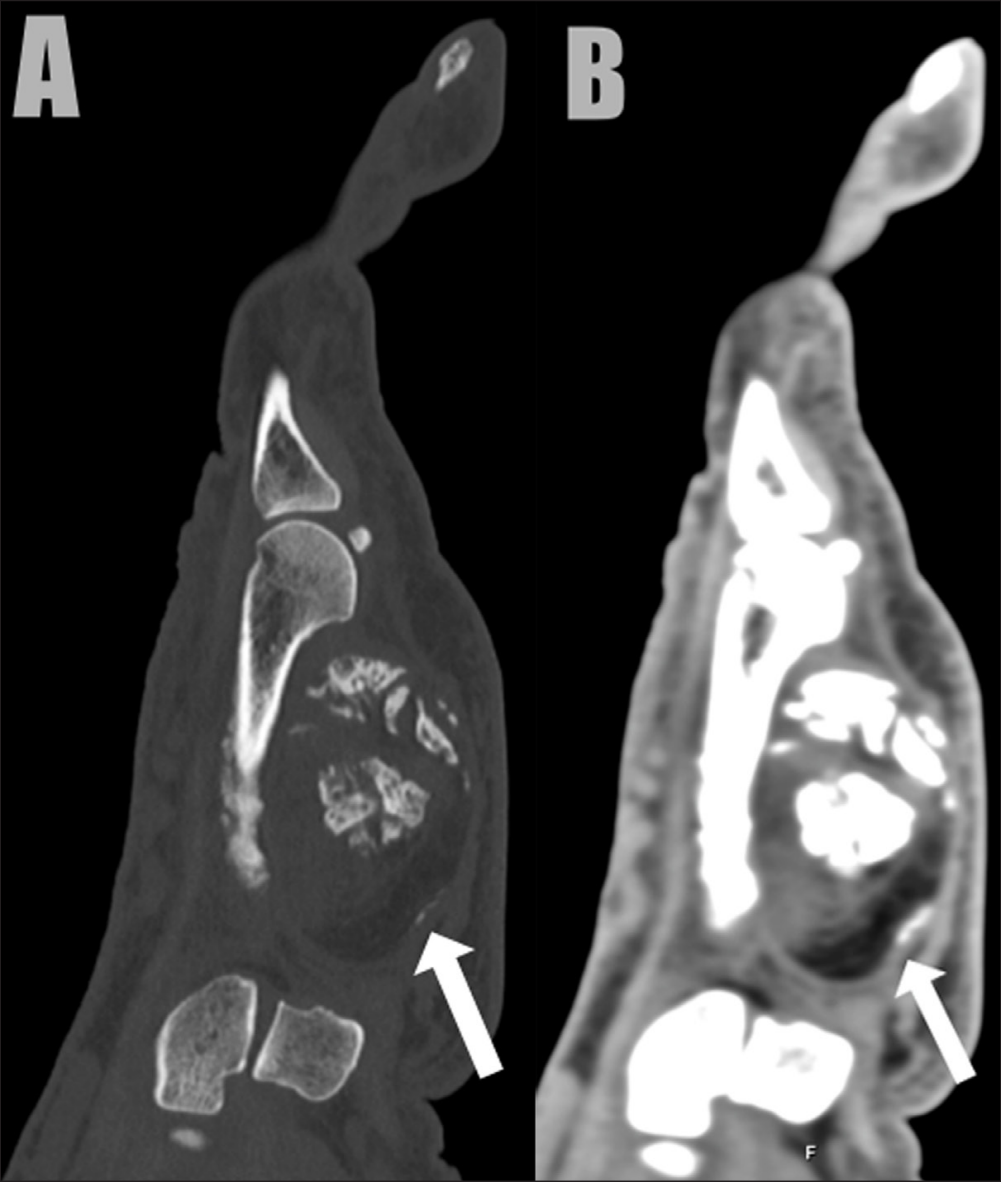


**Figure 2:**
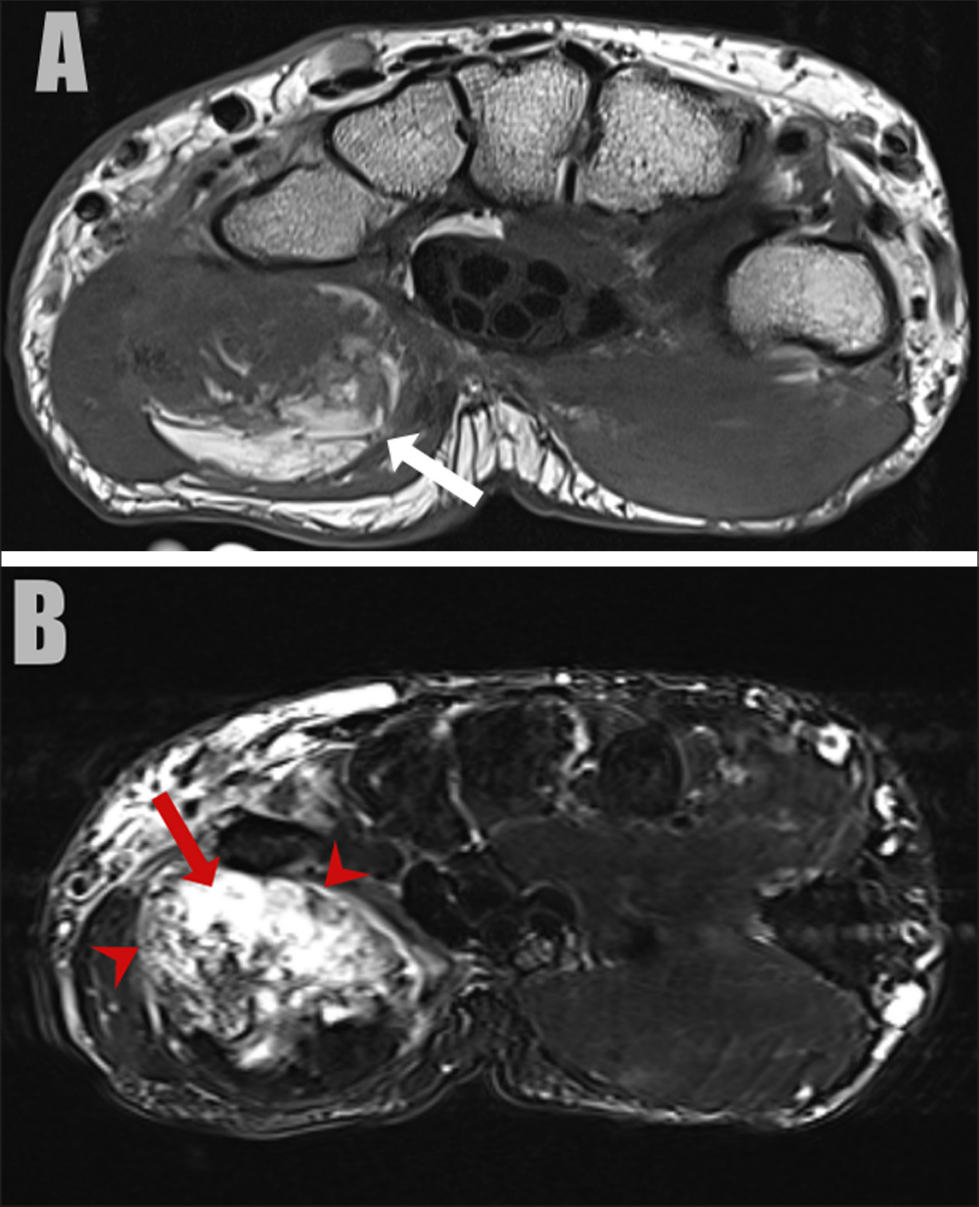


**Figure 3:**
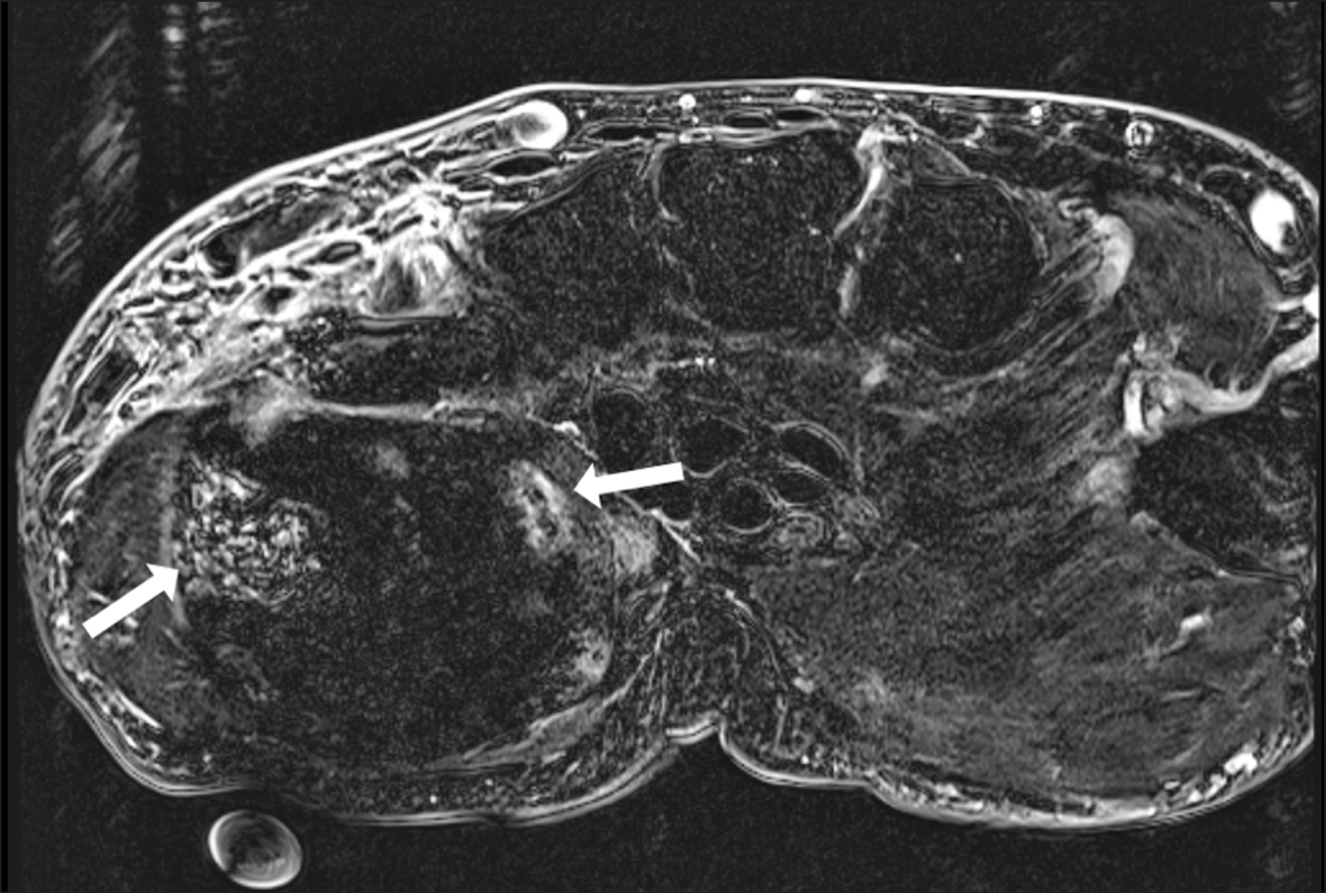


A 79-year-old man presented with a painful swelling in the left hypothenar region, that he noticed for two weeks. Computed tomography (CT) revealed a well-circumscribed mass, located in the hypothenar muscles. The lesion was heterogeneous, containing arciform calcifications, ossifications, and areas of fat and soft tissue density (***Figure 1A–B***: sagittal bone and soft tissue window, arrow). There was no invasion of the lesion in the surrounding structures. Subsequent magnetic resonance imaging (MRI) of the left hand was performed for further grading, characterization, and defining the local extent. MRI confirmed a heterogeneous soft tissue mass located in the adductor pollicis muscle. On the palmar side, the lesion was of high signal intensity on T1-weighted images (WI) in keeping with fat (***Figure 2A***, arrow). In the deep part, foci of low and high signal were seen on fat-suppressed T2-WI (***Figure 2B***, arrowhead and white arrow, respectively). Suppression of the fat signal was seen at the palmar part of the lesion. The areas of low signal correlate with calcifications on CT, whereas the areas of high signal are suggestive for chondromyxoid matrix. The lesion showed delayed and weak enhancement at the periphery of the mass (***Figure 3***, subtraction image, arrows). The diagnosis of a chondroid lipoma was suggested. Because a malignant lesion could not be excluded, the lesion was resected. Histopathological examination confirmed the diagnosis of a chondroid lipoma.

## Comment

A chondroid lipoma is a rare benign adipocytic neoplasm, that presents as a well-circumscribed lesion located in the subcutaneous tissue, superficial muscle fascia or skeletal muscle. It is mostly located in the proximal extremities and has a female predilection. Patients mostly present with a painless, slowly growing lump. Cytogenetic aberrations have been reported associated with a translation between chromosome 11 and 16. Macroscopically, it contains a mixture of fat, chondromyxoid matrix, and metaplastic calcifications or/and ossifications.

Imaging reflects the macroscopic appearance of the lesion. Arciform calcifications, due to calcified chondroid matrix, are usually seen on radiographs and CT. On MRI, calcifications are of low signal on all pulse sequences, whereas non calcified chondromyxoid matrix is of high signal on T2-WI. Fatty components are isointense to subcutaneous fat [1]. The combination of the fatty nature, chondroid-like calcifications on CT and chondromyxoid matrix on MRI is the clue to the correct diagnosis.However, due to the heterogeneity of the lesion, malignancy cannot be excluded on imaging. Therefore, biopsy and histopathological examination is mandatory.
